# Effect of information awareness on attitudes toward human papillomavirus vaccination intentions in Japan

**DOI:** 10.1016/j.jvacx.2024.100599

**Published:** 2024-12-10

**Authors:** Takayuki Takahashi, Takahiro Kinoshita, Daisuke Shigemi, Yousuke Imanishi, Masahiko Sakamoto, Megumi Ichimiya, Makiko Mitsunami, Mihyon Song, Kanako Inaba

**Affiliations:** a Department of Obstetrics and Gynecology, Federation of National Public Service Personnel Mutual Aid Associations, Tachikawa Hospital, Tokyo, Japan; b Minpapi Association, Tokyo, Japan; c Division of General Internal Medicine and Health Services Research, David Geffen School of Medicine at UCLA, Los Angeles, California, USA; d Department of Pediatrics, Saku Central Hospital, Nagano, Japan; e Department of Prevention and Community Health, George Washington University, Washington, DC, USA; f Department of Environmental Health, Harvard T. H. Chan School of Public Health, Boston, USA; g Marunouchi no Mori Ladies Clinic, Tokyo, Japan; h Department of Obstetrics and Gynecology, Kanto Central Hospital, Tokyo, Japan

**Keywords:** Cervical cancer, Human papillomavirus, HPV vaccination, Perception

## Abstract

**Background:**

The Human Papillomavirus (HPV) vaccination rate among Japanese high school girls remains critically low, reflecting ongoing public apprehension and misinformation. This study explores the relationship between information presentation and attitudes toward HPV vaccination in Japan.

**Methods:**

We conducted a web-based survey of female high school students aged 15 to 16 and mothers of daughters of similar age across Japan. The first screening questionnaire investigated the vaccine uptake among eligible students. The detailed questions assessing awareness of HPV vaccination information, including vaccine eligibility and its cost, effectiveness, lifetime prevalence of HPV infection, and vaccine safety, were asked to the respondents. After presenting each piece of information, we investigated how the information influenced the vaccination attitudes of unvaccinated students and mothers of unvaccinated girls.

**Results:**

Data collection occurred from August 20 to August 24, 2021. Of 473 students, 68 (14.4 %) had already been vaccinated before the study. Over half of the 245 participating students were aware of vaccine eligibility and cost (68.7 %) and effectiveness (63.6 %), but fewer understood lifetime prevalence (28.7 %) and safety (45.3 %). In contrast, awareness of the same questions in 245 mothers was higher than that in the students: 90.2 %, 92.5 %, 55.4 %, and 61.1 %, respectively. Among unvaccinated students and mothers of unvaccinated girls, the intention to get vaccinated increased the most after being informed about the lifetime risks of HPV infection; 50.5 % of students and 38.8 % of mothers showed a positive attitude toward the HPV vaccination.

**Conclusions:**

Bridging the awareness gap between students and mothers is crucial for improving HPV vaccination rates. The findings underscore the potential of specific, targeted information to influence vaccination intentions, particularly regarding the HPV infection rate.

## Highlights

• High HPV infection awareness boosted vaccination intentions among students (50.5 %) and mothers (38.8 %).
• Awareness gaps between students and mothers showed the need for targeted educational interventions.
• Vaccine safety and effectiveness information increased vaccination intentions, especially among the unvaccinated.
• Students preferred schools/social media as a source of HPV information; mothers preferred TV/medical institutions.
• Positive general vaccine attitudes were higher among vaccinated individuals, boosting vaccine acceptance.

## 1 Introduction

Cervical cancer ranks fourth in incidence and mortality among cancers affecting women [1]. According to the World Health Organization (WHO), approximately 570,000 people worldwide are diagnosed with cervical cancer each year, of which about 310,000 die from the disease annually [2]. Persistent infection with Human papillomavirus (HPV) is the leading cause of cervical cancer [3]. While HPV vaccination is widely recognized in official guidelines as a safe and effective measure in preventing cervical cancer in Japan [4,5], public perception often diverges, making it essential for government bodies, healthcare providers, and educational institutions to understand the characteristics of both the “vaccinated generation” and the “unvaccinated generation” and to provide accurate and effective educational efforts to promote vaccination [6].

Japan approved the HPV vaccine in 2009 and introduced it into the national routine immunization program in 2013. This introduction was initially successful, with vaccination rates exceeding 70 % among those born between 1994 and 1999, mainly due to robust governmental campaigns and recommendations from healthcare professionals [[7], [8], [9], [10]]. However, in early 2013, Japanese media began disseminating reports of adverse events and unverified risks associated with the HPV vaccine. This negative media controversy significantly influenced public opinion and led the Ministry of Health, Labor, and Welfare, to suspend proactive HPV vaccine recommendations in June 2013 [[11], [12], [13], [14]].

Consequently, HPV vaccination rates plummeted to less than 1 % among those born after 2000 despite substantial evidence supporting the vaccine's safety of the vaccine [9,[15], [16], [17]]. The cessation of recommendations is projected to result in approximately 10,000 additional morbidity cases of cervical cancer between 2020 and 2069 [18], highlighting the urgent need for sustained public health efforts to restore vaccine confidence [14,19].

Several studies have examined the effects of social interventions on HPV vaccination coverage [20,21], with most focusing on mothers [19,22], and only a limited number addressing teenagers themselves in Japan [23]. Factors influencing the willingness to vaccinate include mothers' awareness and knowledge of cervical cancer screening, which has been suggested to be crucial [24], while fathers are less influential [25]. This underscores the importance of educating young females and mothers with children of a similar age about cervical cancer and the HPV vaccine [26]. In our previous study, pediatricians used HPV vaccine safety leaflets to inform mothers, which positively influenced their decision to vaccinate their children [27]. This suggests that providing detailed information about HPV vaccine safety and potential adverse events is essential. However, the specific types of information that contribute most effectively to higher vaccination coverage have not yet been fully explored.

In this study, we aimed to examine the relationship between HPV vaccination coverage and information awareness among 15 to 16-year-old female students and mothers with daughters of similar age in Japan. We investigated how the presentation of various information affected their attitudes and intentions toward HPV vaccination. Specifically, we assessed the impact of information on free vaccination availability, vaccine efficacy, HPV infection rate, and vaccine safety on their vaccination intentions. Additionally, we explored the sources from which participants preferred to receive information about the HPV vaccine, as well as their levels of anxiety and motivations related to vaccination.

## 2 Methods

### 2.1 Study design and participants

This study employed a two-step survey conducted through a third-party company via a web-based questionnaire from August 20, 2021, to August 24, 2021, in Japan. The survey was designed to assess HPV vaccination rates and related factors among two independent groups: first-year female high school students aged 15 to 16 (hereafter referred to as “students”) and mothers of daughters of a similar age in their first year of high school (hereafter referred to as “mothers”). The initial screening survey was distributed nationwide, including 473 students and 326 mothers. Among the 473 students, 68 (14.4 %) of the students had already been vaccinated against the HPV vaccine before participating in the survey.

From the screening survey participants, 245 students and an equal number of mothers consented to participate in the detailed survey, resulting in 490 respondents. Among these 245 students, 57 (23.3 %) had already been vaccinated against the HPV vaccine. Response weightings were adjusted to align the vaccination rate in the detailed survey cohort with the rate observed in the initial screening survey (14.4 %). Specifically, the number of vaccinated students was reduced to 35, with a weighting factor of 0.614, while the number of unvaccinated students was increased to 210, with a weighting factor of 1.117. These groups were not paired as parent-child pairs.

### 2.2 Survey questionnaire

The screening survey consisted of 13 items, including essential attributes (gender, age, residence), awareness of the existence of the HPV vaccine, vaccination status, and vaccination intention among non-vaccinated individuals. Additionally, mothers were asked about their children's vaccination status.

The detailed survey comprised 34 items and was structured to examine various factors associated with HPV vaccination behaviors. The questionnaire addressed factors such as awareness and sources of specific information (free vaccination eligibility, efficacy, lifetime prevalence, and safety), vaccination intentions after information presentation, reasons for non-vaccination, anxiety levels at vaccination, and factors overcoming vaccination anxiety.

Questions also covered general attitudes toward vaccinations, awareness of HPV vaccine facts, and intentions to vaccinate. Additionally, participants were asked about preferred sources and media for obtaining HPV vaccine information, including social networking services (SNS). Open-ended questions were also included to gather reasons for non-vaccination from non-vaccinated individuals and to understand reasons for non-vaccination after receiving the provided information.

### 2.3 Study procedures

The survey presented participants with specific information about the HPV vaccine, followed by questions assessing their awareness of each fact. The information provided was as follows:

① Vaccination eligibility and its cost: HPV vaccination is available free of charge for girls from sixth grade in elementary school to first-year high school students.
② Effectiveness: The HPV vaccine has been reported effective in preventing cervical cancer.
③ Lifetime prevalence: Approximately 80 % of individuals are reported to be infected with HPV, the virus responsible for causing cervical cancer, at least once in their lifetime.
④ Safety: The HPV vaccine is reported to have a safety profile comparable to other routine childhood vaccinations.

These information items were presented sequentially in the following order: vaccination eligibility and cost, effectiveness, lifetime prevalence, and safety. After each piece of information was given, participants were asked to reassess their intentions to receive the HPV vaccine. Vaccination intention was evaluated using a five-point scale, with the response options being “strongly agree,” “somewhat agree,” “neutral,” “somewhat disagree,” and “strongly disagree.”

Finally, unvaccinated students and mothers of unvaccinated students were asked to identify which of the following information items they found most influential in shaping their attitudes toward HPV vaccination: effectiveness, lifetime prevalence, or safety.

### 2.4 Statistics

Simple tabulation was used to calculate the number and percentage of responses.

### 2.5 Ethical considerations

The study was reviewed and approved by the Institutional Review Board at Kanto Central Hospital of the Mutual Aid Association of Public School Teachers (R6–2). Informed consent was waived due to the nature of the study, which involved the retrospective analysis of de-identified data. Information about the study was posted on the hospital's website to provide participants with the opportunity to opt-out.

## 3 Results

### 3.1 General attitudes toward vaccines

In the vaccinated group, the average age was 15.5 for students and 46.47 for mothers, while in the unvaccinated group, the average age was also 15.5 for students and 46.53 for mothers. 82.2 % of the students were aware of the HPV vaccine, compared to a higher awareness rate of 98.6 % among mothers.

**Fig. 1:**
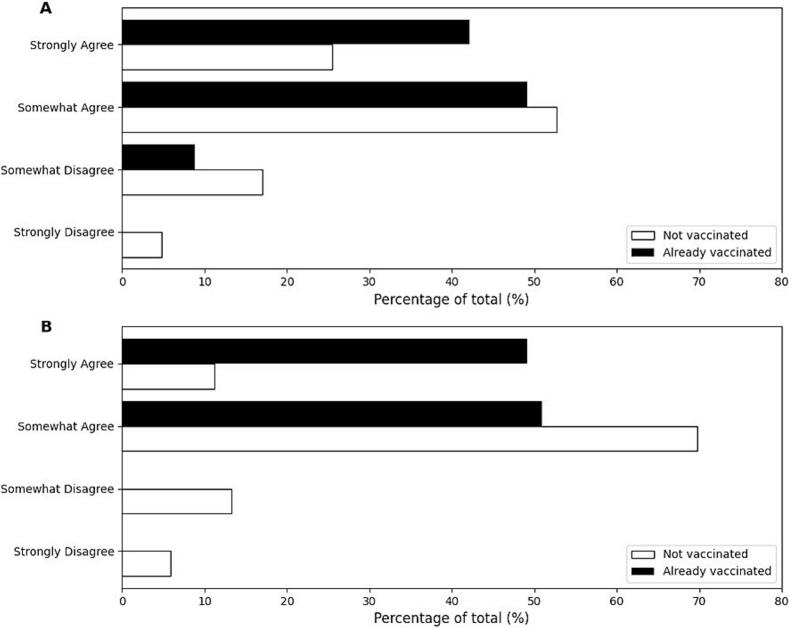
General attitudes toward overall vaccines based on HPV vaccination status. A: General attitudes toward overall vaccines among female high school students. B: General attitudes toward overall vaccines among mothers with children of a similar age. Black bars: Already vaccinated group; White bars: Not vaccinated group.

Fig. 1 shows the results of evaluating their general attitudes toward overall vaccines to understand the characteristics of the study participants (students and mothers).

Among female high school students, the overall attitudes toward vaccines were predominantly positive. Fifty-two point two % of the students agreed somewhat, and 27.9 % strongly agreed with positive attitudes toward vaccines. When analyzed by vaccination status, 52.7 % of the unvaccinated students somewhat agreed, and 25.5 % strongly agreed (Fig. 1A). In contrast, 49.1 % somewhat agreed, and 42.1 % strongly agreed with the vaccinated students. The proportion of unvaccinated students who somewhat disagreed or strongly disagreed was 17.0 % and 4.8 %, respectively, whereas these proportions were lower among vaccinated students at 8.8 % and 0.0 %, respectively (Fig. 1A).

Similarly, mothers also demonstrated positive attitudes toward vaccines. Among the mothers, 67.0 % somewhat agreed, and 16.6 % strongly agreed with positive attitudes toward vaccines. When analyzed by their children's vaccination status, 69.7 % of mothers of unvaccinated students somewhat agreed, and 11.2 % strongly agreed (Fig. 1B). Among the mothers of vaccinated students, 50.9 % somewhat agreed, and 49.1 % strongly agreed (Fig. 1B). The proportion of mothers of unvaccinated students who disagreed somewhat or strongly disagreed was 13.3 % and 5.9 %, respectively, whereas no vaccinated students reported disagreement (Fig. 1B).

### 3.2 Baseline attitudes toward HPV vaccination before information presentation

**Fig. 2:**
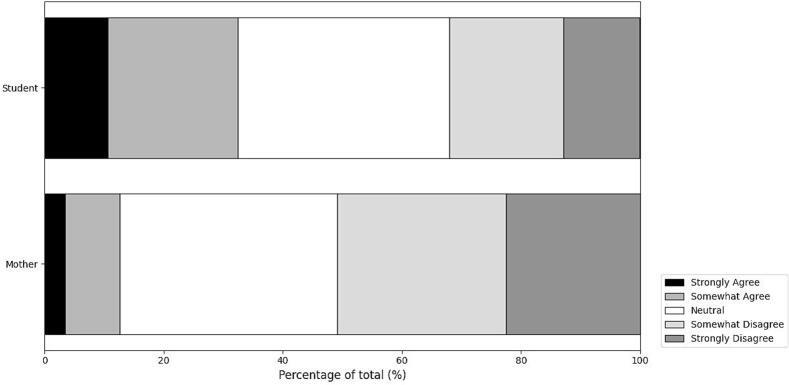
HPV vaccination intentions among unvaccinated individuals. A: Female high school students. B: Mothers with children of a similar age. Attitudes toward HPV vaccination are represented in grayscale: Black: Strongly Agree; Dark Gray: Somewhat Agree; White: Neutral; Light Gray: Somewhat Disagree; Gray: Strongly Disagree.

Fig. 2 depicts the baseline attitudes toward HPV vaccination among unvaccinated female high school students and mothers before any information presentation.

Among unvaccinated female high school students, the majority expressed either neutral or positive attitudes toward HPV vaccination. Expressly, 10.6 % of the students strongly agreed with receiving the HPV vaccine, while 21.8 % somewhat agreed, resulting in 32.4 % with positive attitudes. Conversely, 35.6 % of the students expressed a neutral stance. Negative attitudes were observed in 19.1 % who somewhat disagreed and 12.8 % who strongly disagreed, totaling 31.9 % with negative attitudes (Fig. 2A).

Among mothers of unvaccinated students, the attitudes were predominantly neutral or negative. Only 3.4 % of the mothers strongly agreed, and 9.2 % somewhat agreed, resulting in 12.6 % with positive attitudes. A significant proportion of mothers (36.5 %) expressed neutral attitudes. Negative attitudes were more prevalent, with 28.4 % somewhat disagreeing and 22.5 % strongly disagreeing, resulting in a total of 50.9 % with negative attitudes (Fig. 2B).

These baseline findings highlight the initial perspectives of unvaccinated individuals and establish a reference score for assessing how subsequent information influences attitudes in these groups.

### 3.3 Association between awareness of each information and HPV vaccination status

Among all students, over half were aware of the information regarding vaccine eligibility and cost (68.7 %) and effectiveness (63.6 %), In contrast, awareness levels were lower for lifetime prevalence (28.7 %) and safety (45.3 %). Mothers exhibited higher awareness across all four categories, with 90.2 %, 92.5 %, 55.4 %, and 61.1 %, respectively.

**Fig. 3:**
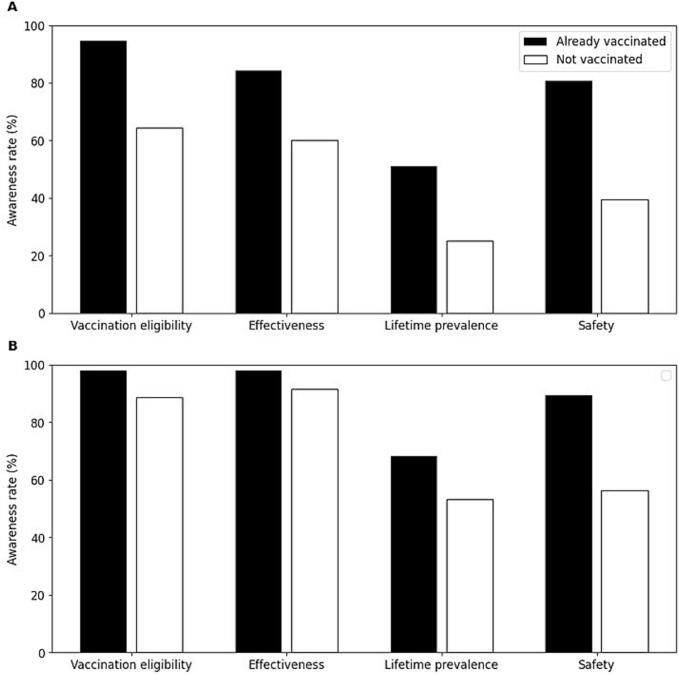
Awareness rate of HPV vaccine-related information based on HPV vaccination status. A: Awareness rate among female high school students. B: Awareness rate among mothers with children of a similar age. The awareness rates for four key information points (vaccination eligibility, effectiveness, lifetime prevalence, and safety) are shown. Black bars: Already vaccinated group; White bars: Not vaccinated group.

For students already vaccinated against HPV, the awareness rate for vaccination eligibility and its cost, effectiveness, lifetime prevalence, and safety were 94.7 %, 84.2 %, 50.9 %, and 80.7 %, respectively (Fig. 3A, black). Conversely, for students not vaccinated against HPV, the awareness rate for these same categories was lower, with 64.4 %, 60.1 %, 25.0 %, and 39.4 %, respectively (Fig. 3A, White). The differences in awareness between vaccinated and unvaccinated students for the four pieces of information were as follows: vaccination eligibility and its cost (30.3 %), effectiveness (24.1 %), lifetime prevalence (25.9 %), and safety (41.3 %).

Among mothers of vaccinated children, the awareness rates for information regarding vaccination eligibility and its cost, effectiveness, lifetime prevalence, and safety were 98.2 %, 98.2 %, 68.4 %, and 89.5 %, respectively (Fig. 3B, black). In contrast, the mothers of unvaccinated children showed lower awareness levels for the same categories: 88.8 %, 91.5 %, 53.2 %, and 56.4 %, respectively (Fig. 3B, white). The differences in awareness between these groups of mothers were vaccination eligibility and its cost (9.4 %), effectiveness (6.7 %), lifetime prevalence (15.2 %), and safety (33.1 %).

### 3.4 Effect of information presentation on attitudes toward vaccination intentions among initially unaware respondents

**Fig. 4:**
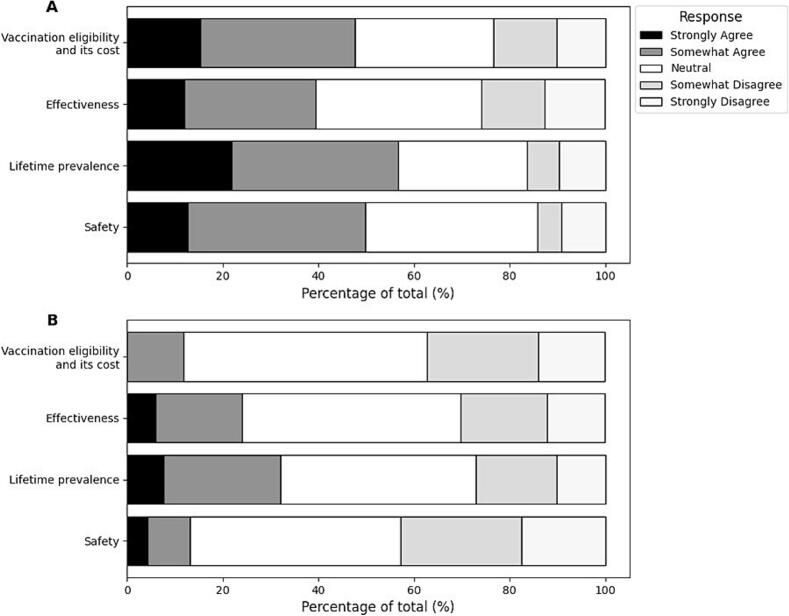
Attitudes toward HPV vaccination after each information presentation. A: Female high school students who were unaware of the information before its presentation. B: Mothers with children of a similar age who were unaware of the information before its presentation. The bar charts illustrate the percentage of respondents' attitudes toward HPV vaccination after receiving information on vaccination eligibility, cost, effectiveness, lifetime prevalence of HPV infection, and safety. Black bars: Strongly Agree; Dark gray bars: Somewhat Agree; White bars: Neutral; Light gray bars: Somewhat Disagree; Gray bars: Strongly Disagree.

Fig. 4 presents the attitudes of students (A) and mothers (B) toward HPV vaccination after various information was presented to them, who were initially unaware of each information.

Among students unaware of vaccination eligibility and its cost, 15.4 % strongly agreed, and 32.2 % somewhat agreed, resulting in 47.6 % with positive attitudes. Neutral responses accounted for 29.1 %, while negative attitudes (somewhat and strongly disagreeing) were observed in 13.1 % and 10.2 %, respectively.

For students initially unaware of the information about effectiveness, 11.9 % strongly agreed and 27.6 % somewhat agreed, resulting in 39.5 % with positive attitudes. Neutral responses were higher at 34.7 %, while 13.2 % somewhat disagreed and 12.5 % strongly disagreed, totaling 25.7 % with negative attitudes.

Presenting lifetime prevalence information elicited the most positive responses among students, with 21.9 % strongly agreeing and 34.8 % somewhat agreeing, totaling 56.7 %. Neutral responses accounted for 27.0 %, while negative attitudes were lower, with 6.7 % somewhat disagreeing and 9.6 % strongly disagreeing, totaling 16.3 %.

For students unaware of the safety information, 12.7 % strongly agreed, and 37.2 % somewhat agreed, resulting in 49.9 % with positive attitudes. Neutral responses accounted for 36.0 %, while 5.0 % somewhat disagreed and 9.2 % strongly disagreed, totaling 14.2 % with negative attitudes.

Among mothers initially unaware of vaccination eligibility and its cost, no respondents strongly agreed, but 11.8 % somewhat agreed, totaling 11.8 % with positive attitudes. Neutral responses dominated at 51.0 %, while 23.2 % somewhat disagreed and 13.9 % strongly disagreed, totaling 37.1 % with negative attitudes.

For mothers unaware of effectiveness, 6.0 % strongly agreed, and 18.1 % somewhat agreed, resulting in 24.1 % with positive attitudes. Neutral responses accounted for 45.6 %, while 18.1 % somewhat disagreed and 12.1 % strongly disagreed, totaling 30.2 % with negative attitudes.

The information about lifetime prevalence, presented to unaware mothers, resulted in 7.7 % strongly agreeing and 24.4 % somewhat agreeing, totaling 32.1 % with positive attitudes. Neutral responses were 40.8 %, while 16.9 % somewhat disagreed and 10.2 % strongly disagreed, totaling 27.1 % with negative attitudes.

Finally, for safety, mothers who were initially unaware demonstrated 4.3 % strongly agreeing and 8.9 % somewhat agreeing, resulting in 13.2 % with positive attitudes. Neutral responses accounted for 44.0 %, while negative attitudes were notably higher, with 25.3 % somewhat disagreeing and 17.6 % strongly disagreeing, totaling 42.9 %.

These findings indicate that among students, the lifetime prevalence of HPV infection information generated the most positive attitudes, while information about safety elicited mixed responses with relatively high neutrality. For mothers, neutral and negative attitudes were predominant across all information categories, particularly for safety, which yielded the highest negative response rates.

### 3.5 Subjective perceptions of the most influential information contributing to vaccination intentions

**Fig. 5:**
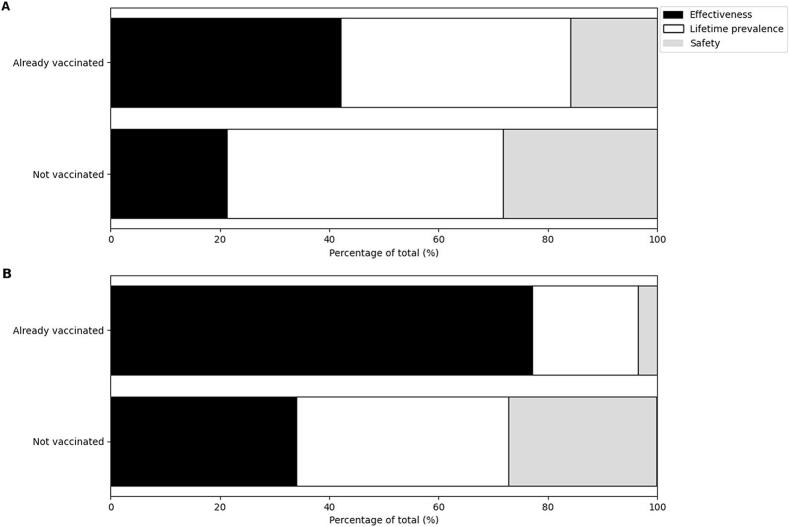
Subjective perceptions of the most influential information contributing to HPV vaccination intention. A: Female high school students. B: Mothers with children of a similar age. The bar charts represent participants' subjective perceptions regarding which information (effectiveness, lifetime prevalence of HPV infection, or safety) most influenced their intention to receive the HPV vaccine. Black bars: Effectiveness; White bars: Lifetime prevalence; Gray bars: Safety.

Students (Fig. 5A) and mothers (Fig. 5B), divided by their vaccination status (for students) and their children's vaccination status (for mothers), were asked to subjectively identify the information they found most influential in shaping their attitudes toward HPV vaccination. This assessment was limited to three specific categories of information: vaccine effectiveness, lifetime prevalence of HPV infection, and vaccine safety.

Among unvaccinated students, 21.3 % selected vaccine effectiveness as the most influential information. A majority, 50.5 %, indicated that the lifetime prevalence of HPV infection was the most compelling, while 28.2 % identified vaccine safety as the most impactful factor. In contrast, vaccine effectiveness was the most commonly selected category for vaccinated students, with 42.1 % identifying it as the most influential information, followed equally by the lifetime prevalence of HPV infection (42.1 %) and vaccine safety (15.8 %).

Among mothers of unvaccinated children, the most commonly selected factor was the lifetime prevalence of HPV infection (38.8 %), followed by vaccine effectiveness (34.0 %) and vaccine safety (27.1 %). In contrast, mothers of vaccinated children overwhelmingly identified vaccine effectiveness as the most influential information, with 77.2 % selecting it. In comparison, smaller proportions chose the lifetime prevalence of HPV infection (19.3 %) and vaccine safety (3.5 %).

These results highlight a stark difference between the groups. For vaccinated individuals (students and mothers), vaccine effectiveness was the most influential information. On the other hand, unvaccinated individuals (students and mothers of unvaccinated children) tended to prioritize the lifetime prevalence of HPV infection as the most compelling factor, with vaccine safety also playing a significant role.

### 3.6 Levels of anxiety and reasons for vaccination

**Fig. 6:**
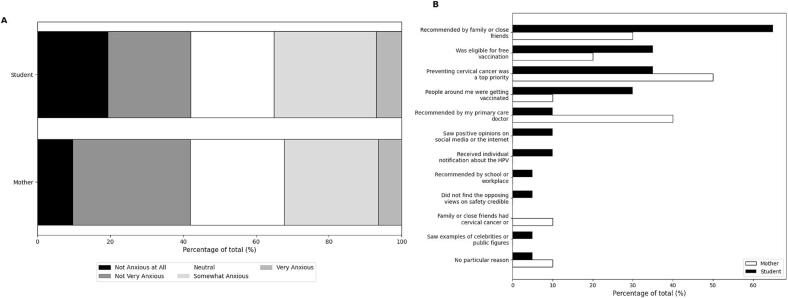
Levels of anxiety at the time of HPV vaccination and reasons for receiving the HPV vaccine. A: Anxiety levels at the time of HPV vaccination among female high school students (Student) and mothers with children of a similar age (Mother). The bar chart shows the percentage distribution of respondents by their level of anxiety at the time of vaccination: Black bars: Not Anxious at All; Dark gray bars: Not Very Anxious; White bars: Neutral; Light gray bars: Somewhat Anxious; Gray bars: Very Anxious. B: Reasons for receiving the HPV vaccine. The horizontal bar chart illustrates the percentage of respondents as reasons to vaccinate. Black bars: Female high school students; White bars: Mothers with children of a similar age.

Fig. 6 presents the levels of anxiety at the time of HPV vaccination and the reasons for vaccination among students and mothers.

Fig. 6A shows the levels of anxiety among students and mothers at the time of HPV vaccination. Among the students, 19.3 % reported not being anxious, 22.8 % were not very anxious, 22.8 % felt neutral, 28.1 % were somewhat anxious, and 7.0 % were very anxious. In contrast, among the mothers, 9.7 % reported not being anxious, 32.3 % were not very anxious, 25.8 % felt neutral, 25.8 % were somewhat anxious, and 6.5 % were very anxious.

The reasons for vaccination among students and mothers are presented in Fig. 6B. For the students, the most common reason for getting vaccinated was a recommendation by family or close friends (65.0 %), followed by being eligible for free vaccination (35.0 %) and the priority of preventing cervical cancer (35.0 %). Other reasons included seeing people around them getting vaccinated (30.0 %), recommendation by their primary care doctor (10.0 %), and upbeat opinions on social media or the internet (10.0 %). For the mothers, the most common reason for vaccinating their daughters was the priority of preventing cervical cancer (50.0 %), followed by recommendations from their primary care doctor (40.0 %) and eligibility for free vaccination (20.0 %). Other reasons included seeing people around them getting vaccinated (10.0 %) and knowing family or close friends who had cervical cancer or precancerous cervical lesions (10.0 %).

### 3.7 Desired sources of information on the HPV vaccine

**Fig. 7:**
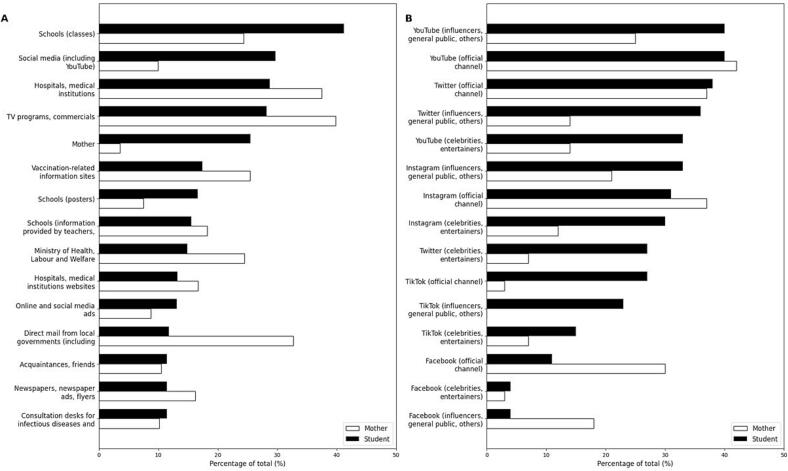
Preferred sources of information about the HPV vaccine. A: Preferred traditional and institutional information sources for HPV vaccine-related information. The bar chart shows the percentage of female high school students (black bars) and mothers with children of a similar age (white bars) who identified specific sources as desirable for obtaining HPV vaccine information. B: Preferred social media platforms for HPV vaccine-related information. The bar chart illustrates the percentage of female high school students (black bars) and mothers with children of a similar age (white bars) who preferred social media platforms, categorized by type as their desired sources of HPV vaccine information.

Fig. 7 presents the preferred sources of information about the HPV vaccine among female high school students and mothers.

Fig. 7A illustrates the preferred sources of information for students and mothers. Among the students, the most preferred source of information was schools, specifically classes (41.2 %), followed by social media, including YouTube (29.7 %), Hospitals/Medical institutions (28.8 %), and TV programs/Commercials (28.2 %). In contrast, among the mothers, the most preferred source of information was TV programs/Commercials (39.9 %), followed by hospitals/medical institutions (37.5 %) and Vaccination-related information sites (25.4 %).

The preferred social media platforms for obtaining HPV vaccine information are shown in Fig. 7B. Among the students, YouTube (influencers, general public, others) was the most preferred platform (40.0 %), followed by YouTube (official channel) (39.9 %), Twitter (official channel) (38.0 %), Twitter (influencers, general public, others) (35.6 %) and YouTube (celebrities, entertainers) (32.7 %). For mothers, YouTube (official channel) was the most preferred platform (42.0 %), followed by Twitter (official channel) (37.1 %), Instagram (official channel) (37.1 %), Facebook (official channel) (30.0 %), and YouTube (influencers, general public, others) (25.4 %). The medium with the most significant difference between students and mothers was TikTok (official channel) (24.0 %), with mothers having the lowest percentage.

## 4 Discussion

This study, focusing on a specific cohort, found that among first-year high school girls, the HPV vaccination rate was significantly low at 14.3 %, highlighting a sharp decline from the over 70 % vaccination rate observed during active vaccine promotion (2010−2013). Despite the resumption of active HPV vaccine recommendations in 2022 [28], public concerns and misunderstandings about the vaccine's safety and efficacy persist [6]. Inadequate and inaccurate information, fueled by improper their media reporting, exacerbates anxiety and hesitation toward vaccination. Therefore, the Ministry of Health, Labour and Welfare must adopt a behavioral economics approach, strengthen media strategies, and provide safe, scientifically based information to the public in Japan [29]. Similar challenges have been noted in high-income countries like France, where misinformation and insufficient communication have been identified as critical barriers to vaccine uptake and are often exacerbated by inappropriate media coverage [30]. Such barriers highlight the urgent need for targeted, evidence-based strategies to rebuild trust and address vaccine hesitancy [31].

### 4.1 Disparities in awareness and the impact of information on vaccination intentions

The survey demonstrated significant disparities in the awareness of HPV vaccine-related information between students and mothers, with students showing deficient awareness of HPV infection rates compared to mothers. Vaccination status further influenced awareness, as vaccinated students demonstrated a higher understanding of HPV-related information than their unvaccinated peers. This aligns with the findings that prior vaccination experiences enhance vaccine-related knowledge and acceptance  [31,32].

Significantly, when comparing baseline attitudes (Fig. 2) with attitudes after information presentation (Fig. 4), none of the provided information types reduced vaccination intentions. On the contrary, all information types increased positive attitudes among students and mothers. Among students, information about the lifetime prevalence of HPV infection elicited the most substantial positive response, significantly increasing vaccination intentions. Similarly, mothers showed the most significant increase in vaccination intentions when presented with information about lifetime prevalence, underscoring the impact of this specific message. These findings emphasize that accurate and targeted information can effectively address hesitancy and enhance vaccine acceptance in both groups.

The data further suggest that the lifetime prevalence of HPV serves as a critical motivator for vaccination. This may be due to its ability to increase perceived susceptibility, which the Health Belief Model points to the perceived likelihood of disease (morbidity) influencing a person's health behavior [33]. These findings reinforce the importance of tailoring public health messages to different demographic groups' specific informational needs and perceptions.

### 4.2 Tailored communication strategies

Our findings also suggest that vaccinated students and mothers tend to have more positive attitudes toward vaccines than those who have not. This indicates a correlation between vaccination status and general attitudes toward vaccines, highlighting the potential for increased vaccination rates to improve vaccine acceptance. Moreover, different types of information have varying impacts on the vaccination intentions of students and mothers. Information about the lifetime prevalence of HPV had the most substantial positive effect on students, while details about vaccination eligibility and its cost were most influential for mothers. This indicates the importance of tailored communication strategies to enhance HPV vaccination rates among target groups. These findings are consistent with prior research in Canada, where targeted campaigns tailored to demographic-specific concerns significantly enhanced vaccination rates [32].

### 4.3 Addressing vaccine hesitancy and improving uptake

To enhance the HPV vaccination rate, it is essential to provide comprehensive education about cervical cancer and screening to both mothers and daughters, not just the vaccine provision in Japan. The study highlights key factors influencing vaccination decisions. Recommendations from family and friends were particularly influential for students, while mothers prioritized medical recommendations and the prevention of cervical cancer. These findings align with trends observed in Central Asia, where social and familial influences significantly impacted vaccine uptake [34]. Understanding these motivations is crucial for designing interventions that address specific concerns and priorities for different groups. Dionne et al. (2024) demonstrated the effectiveness of such multimodal strategies in improving vaccination rates [32]. However, the accuracy and quality of information, particularly regarding vaccine safety, remain paramount. As Yagi et al. (2024) cautioned, insufficient explanations of vaccine safety may perpetuate parental anxiety, underscoring the need for transparent, evidence-based communication [14].

Educational campaigns incorporating diverse approaches, including leaflets, smartphone apps [35], and games [36,37] can further enhance vaccine acceptance. However, the quality of information provision, especially regarding the safety of the HPV vaccine, remains a concern. The lack of detailed safety explanations may contribute to parental anxiety and low vaccination rates.

### 4.4 Implications for healthcare providers

Healthcare providers, particularly pediatricians, are critical in addressing parental concerns and promoting HPV vaccination. However, as Katsuta et al. (2021) noted, many pediatricians in Japan feel less confident than OB/GYNs in educating parents about HPV-related sexually transmitted infections [38]. Enhancing the training and support available to pediatricians can empower them to provide accurate and reassuring information, highlighting the critical role of healthcare provider recommendations in overcoming vaccine hesitancy [30,34]. Pediatricians play a crucial role in building trust with parents and children [[39], [40], [41]]. Collaborative Initiatives, like the “Minpapi! Everyone's HPV Project” implemented by non-governmental organizations and proactive information dissemination by pediatricians may contribute to increased awareness and vaccination rates.

### 4.5 Social media as an outreach platform

Social media platforms like YouTube, Instagram, and Twitter emerged as widely preferred sources of information among students and mothers. Proactive engagement on these platforms can significantly enhance the reach and impact of public health campaigns [31,42]. The study further indicates that YouTube is a widely preferred platform for both students and mothers, though students also strongly prefer Instagram and Twitter. Mothers, on the other hand, favor YouTube and Instagram but are less inclined toward TikTok than students. Overall, the findings emphasize the importance of utilizing diverse information sources and social media platforms to reach different demographics effectively. Utilizing platforms favored by younger audiences, such as Instagram and Twitter for students, alongside YouTube for mothers, can help disseminate accurate HPV vaccine information effectively.

### 4.6 Limitation

The present study is a cross-sectional analysis based on a survey. As such, it has certain limitations, including the potential for selection bias associated with sampling participants and the inability to establish causal relationships.

## 5 Conclusion

In conclusion, this study illuminates the complex challenges surrounding the HPV vaccination in Japan, particularly among first-year high school girls. Despite renewed efforts to promote the vaccine, persistent public concerns and misunderstandings significantly hinder achieving a higher vaccination rate. Addressing these issues requires a multifaceted approach, including tailored public health communication strategies, comprehensive education, and robust support for healthcare providers. Successfully enhancing vaccine uptake will necessitate providing accurate and reassuring information and a deeper understanding of the varied perceptions and attitudes toward vaccination across different demographic groups. In particular, it was discernible from the study that information regarding the HPV infection rate contributed most significantly to the willingness of high school girls to consider HPV vaccination. This finding highlights the critical impact of specific information on shaping vaccination intentions in this demographic.

## Funding

This study was funded by crowdfunding supporters on READYFOR Inc.

## CRediT authorship contribution statement

**Takayuki Takahashi:** Writing – review & editing, Writing – original draft, Visualization, Supervision, Software, Resources, Project administration, Investigation, Funding acquisition, Formal analysis, Conceptualization. **Takahiro Kinoshita:** Writing – review & editing, Supervision, Funding acquisition. **Daisuke Shigemi:** Writing – review & editing, Supervision, Methodology, Formal analysis. **Yousuke Imanishi:** Supervision. **Masahiko Sakamoto:** Writing – review & editing. **Megumi Ichimiya:** Writing – review & editing, Methodology. **Makiko Mitsunami:** Writing – review & editing, Methodology. **Mihyon Song:** Writing – review & editing. **Kanako Inaba:** Writing – review & editing, Project administration.

## Declaration of competing interest

The authors declare the following financial interests/personal relationships which may be considered as potential competing interests: Takayuki Takahashi reports financial support was provided by READYFOR Inc. If there are other authors, they declare that they have no known competing financial interests or personal relationships that could have appeared to influence the work reported in this paper.

## Data Availability

Data will be made available on request.

## Appendix A Supplementary data

Supplementary material Image 1
